# HilD induces expression of a novel *Salmonella* Typhimurium invasion factor, YobH, through a regulatory cascade involving SprB

**DOI:** 10.1038/s41598-019-49192-z

**Published:** 2019-09-04

**Authors:** María M. Banda, Rubiceli Manzo, Víctor H. Bustamante

**Affiliations:** Departamento de Microbiología Molecular, Instituto de Biotecnología, Universidad Nacional Autónoma de México, Cuernavaca, Morelos 62210 Mexico

**Keywords:** Pathogens, Bacteriology

## Abstract

HilD is an AraC-like transcriptional regulator encoded in the *Salmonella* pathogenicity island 1 (SPI-1), which actives transcription of many genes within and outside SPI-1 that are mainly required for invasion of *Salmonella* into host cells. HilD controls expression of target genes directly or by acting through distinct regulators; three different regulatory cascades headed by HilD have been described to date. Here, by analyzing the effect of HilD on the *yobH* gene in *Salmonella enterica* serovar Typhimurium (*S*. Typhimurium), we further define an additional regulatory cascade mediated by HilD, which was revealed by previous genome-wide analyses. In this regulatory cascade, HilD acts through SprB, a LuxR-like regulator encoded in SPI-1, to induce expression of virulence genes. Our data show that HilD induces expression of *sprB* by directly counteracting H-NS-mediated repression on the promoter region upstream of this gene. Then, SprB directly activates expression of several genes including *yobH*, *slrP* and *ugtL*. Interestingly, we found that YobH, a protein of only 79 amino acids, is required for invasion of *S*. Typhimurium into HeLa cells and mouse macrophages. Thus, our results reveal a novel *S*. Typhimurium invasion factor and provide more evidence supporting the HilD-SprB regulatory cascade.

## Introduction

The genus *Salmonella* groups facultative anaerobic Gram-negative bacteria and is divided into two species, *S. enterica* and *S. bongori*. The former is responsible for diseases ranging from gastroenteritis to severe systemic infections in a wide range of hosts, and it comprises 6 subspecies that are further divided into serovars^1,2^. The broad-host-range *S. enterica* serovar Typhimurium (*S*. Typhimurium) is a common cause of gastroenteritis in humans and many animals worldwide; furthermore, it can also cause systemic infection in humans and some animals, including laboratory mice^1,3,4^. For this reason, *S*. Typhimurium is frequently used as a model for studying the host-pathogen interactions during infection with *Salmonella*.

During its evolution, *Salmonella* has acquired numerous DNA fragments through different horizontal transference events^5,6^; those encoding virulence factors are denominated *Salmonella* pathogenicity islands (SPIs)^7–9^. Up to 22 SPIs have been described among *Salmonella* serovars^10^, being SPI-1 and SPI-2 the most importantly involved in gastroenteritis and systemic disease, respectively^2,8,9^. SPI-1 is present in the two *Salmonella* species, in contrast, SPI-2 is present only in the *S. enterica* species, which supports that SPI-1 was acquired earlier than SPI-2 during the *Salmonella* pathogenicity evolution^5,11^.

SPI-1 is a ∼40 kb chromosomal region of *Salmonella* that contains 39 genes, which encode a type III secretion system (T3SS-1; a syringe-like molecular apparatus that extent from the membranes of bacteria), several effector proteins and their respective chaperones, as well as some transcriptional regulators^8,12,13^. Translocation of effector proteins into the cytoplasm of eukaryotic cells, through the T3SS-1, favors invasion of *Salmonella* into these cells by a trigger mechanism, which involves cytoskeletal rearrangements known as “membrane ruffles”^8,12^. Invasion of *Salmonella* into the intestinal epithelium induces strong intestinal inflammatory response leading to gastroenteritis; in turn, this generates different antimicrobial activities that displace most of the intestinal microbiota, which heighten the intestinal colonization by *Salmonella*^2,9,14,15^. Consistently with their role in intestinal disease, the SPI-1 genes are expressed *in vivo* when *Salmonella* resides in the intestinal lumen and in the cytosol of epithelial cells^16,17^. Moreover, expression of the SPI-1 genes is regulated by distinct molecules or conditions present in the intestine of humans and animals, such as short- and long-fatty acids, high osmolarity, bile, low level of aeration and neutral pH^18–23^. In laboratory conditions, the SPI-1 genes are expressed in nutrient-rich media like the lysogeny broth (LB), where they are expressed in the late exponential (early stationary) phase of growth, which somehow mimic the intestinal environment^24–26^.

Expression of the SPI-1 genes is controlled by a myriad of global and *Salmonella*-specific regulators. HilD, an AraC-like transcription regulator encoded within SPI-1, is the apex of different regulatory cascades controlling expression of tens of *Salmonella* virulence genes^2,27,28^. HilD positively regulates expression of the SPI-1 genes and many other related genes encoded outside SPI-1, by inducing expression of HilA, an OmpR-ToxR-like transcriptional regulator, which in turn induces expression of InvF, also an AraC-like transcriptional regulator; HilA and InvF, both encoded in SPI-1, directly induce expression of the rest of SPI-1 genes^2,29^. HilD controls expression of *hilA* directly or through a feed-forward loop that it forms with HilC and RtsA, encoded within and outside SPI-1, respectively; although these three AraC-like regulators recognize the same DNA motif, HilD is dominant over HilC and RtsA^30,31^. On another hand, HilD positively controls expression of the FlhD_4_C_2_ complex, the master positive transcriptional regulator of the flagellar/chemotaxis genes, which are also required for the invasion phenotype of *Salmonella*^32–35^. Likewise, HilD induces expression of the SPI-2 genes, which are mainly required for replication of *Salmonella* within host cells, by acting on the *ssrAB* operon that codes for the SsrA/SsrB two-component system, the master regulator of SPI-2^25,36,37^. Additionally, HilD directly controls expression of several other virulence genes^28,38–40^. For all cases characterized up to now, HilD induces expression of target genes by directly antagonizing repression mediated by the histone-like protein H-NS on the respective promoters^36,37,41–43^.

In addition to HilD, HilC, HilA and InvF, SPI-1 also codes for SprB, which belongs to the LuxR/UhaP family of transcriptional factors^44^. SprB is not required for expression of the SPI-1 genes, but it is expressed under the same conditions that favor expression of these genes^28,44,45^; furthermore, a previous study indicates that expression of SprB is positively regulated by HilA^46^. Firstly, SprB was shown to directly regulate expression of the *siiABCDEF* operon located in SPI-4^46^; however, recent transcriptomic analyses support that SprB controls expression of several virulence and hypothetical genes, but not that of the *siiABCDEF* operon^28^.

Data from this study, together with previous results from genome-wide analyses^28^, define an additional regulatory cascade headed by HilD. In this regulatory cascade, HilD induces expression of SprB, which in turn activates expression of several target genes including *yobH*, *slrP* and *ugtL*; *slrP* and *ugtL* have been involved in *Salmonella* virulence. Our results show that HilD directly induces expression of *sprB* by antagonizing repression mediated by H-NS. Interestingly, we found that the *yobH* gene is required for invasion HeLa cells and mouse macrophages by *S*. Typhimurium, which reveals a novel invasion factor of *Salmonella*. Thus, the HilD-SprB regulatory cascade represents a novel pathway that controls expression of virulence genes in *Salmonella*.

## Results

### HilD positively controls the expression of *yobH* (*SL1344_1770*)

Previous RNA-sequencing (RNA-seq) analysis indicates that the HilD transcriptional regulator positively controls the expression of the *S*. Typhimurium *SL1344_1770* gene^28^, which is located outside SPI-1 and codes for a hypothetical protein of 79 amino acids. Additional RNA-seq and co-expression analyses also support that HilD is involved in the expression of *SL1344_1770*^39,45^. Orthologs of *SL1344_1770*, which show high sequence identity and a conserved genomic context, are denominated *yobH* in *Escherichia coli* and several other bacteria; thus, we kept the name of *yobH* for *SL1344_1770*.

**Figure 1:**
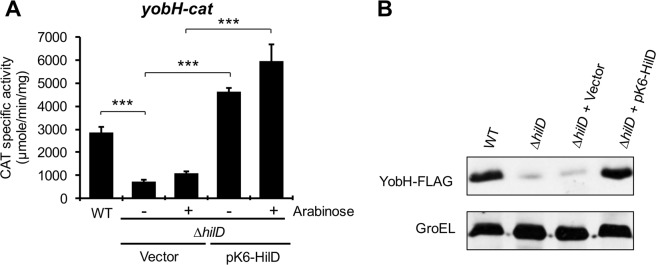
HilD positively regulates the expression of *yobH* (*SL1344_1770*) in LB. (**A**) Activity of the *yobH-cat* transcriptional fusion from the pyobH-cat plasmid, was determined in the WT *S*. Typhimurium SL1344 strain and its isogenic ∆*hilD* mutant containing the pMPM-K6Ω vector or the pK6-HilD plasmid, with (+) and without (−) induction (0.001% L-arabinose). Means and standard deviations from three independent experiments performed in duplicate are shown. Statistically different values are indicated (***p < 0.001). (**B**) Expression of YobH-FLAG in WT *S*. Typhimurium SL1344 strain and its isogenic ∆*hilD* mutant containing the pMPM-K6Ω vector or the pK6-HilD plasmid, was analyzed by Western blotting by using an anti-FLAG monoclonal antibody. GroEL was detected as a loading control with an anti-GroEL polyclonal antibody. Blots were cropped from different parts of the same gel. CAT specific activity and YobH-FLAG expression were determined from samples of bacterial cultures grown for 9 h in LB at 37 °C.

To confirm the regulation of *yobH* by HilD, a transcriptional fusion of the intergenic region upstream of *yobH* to the *cat* (chloramphenicol acetyl transferase) reporter gene was constructed in the pKK232-8 plasmid. Specific activity from this fusion was quantified in the wild type (WT) *S*. Typhimurium strain SL1344 and its derivative ∆*hilD* mutant, grown in nutrient-rich lysogeny broth (LB) at 37 °C, conditions that induce the expression of genes regulated by HilD^25,26,39^. The activity of the *yobH-cat* fusion showed a 3-fold reduction in the ∆*hilD* mutant, compared with its expression in the WT strain; in addition, the expression of HilD from the pK6-HilD plasmid increased around 5-fold the activity of this fusion in the ∆*hilD* mutant (Fig. 1A). To investigate if *yobH* indeed codes for a protein and to further confirm the positive regulation of *yobH* by HilD, we tested the expression of the YobH-FLAG putative protein (YobH tagged with a 3XFLAG epitope) in the WT *S*. Typhimurium strain and its derivative ∆*hilD* mutant. A specific signal for YobH-FLAG was detected in the WT strain, with the expected size for this protein (Fig. 1B). The amount of YobH-FLAG was almost abolished in the ∆*hilD* mutant; as expected, it was restored at WT levels by the expression of HilD from the pK6-HilD plasmid (Fig. 1B). Taken together, these results show that HilD induces the expression of *yobH*.

### YobH is involved in the *S*. Typhimurium invasion of host cells

**Figure 2:**
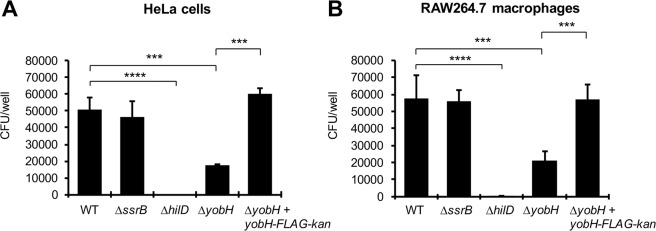
YobH is involved in the *S*. Typhimurium invasion of HeLa cells and macrophages. Epithelial HeLa cells (**A**) and murine RAW 264.7 macrophages (**B**) were infected with the WT *S*. Typhimurium SL1344 strain and its isogenic ∆*ssrB*, ∆*hilD*, ∆*yobH* and ∆*yobH* + *yobH-FLAG-kan* (∆*yobH* complemented) mutants. Invasion was quantified by enumerating the intracellular CFUs at 1 h post-infection, using a gentamicin protection assay. Means and standard deviations from three independent experiments performed in duplicate are shown. Statistically different values are indicated (***p < 0.001; ****p < 0.0001).

HilD positively regulates expression of numerous genes mainly required for the invasion of *Salmonella* into host cells^2,28,39,45^. For instance, recently we found that HilD controls expression of the *grhD1* invasion gene, which is located outside SPI-1^40^. To determine whether YobH is required for this *Salmonella* virulence phenotype, we evaluated the invasion ability of the WT *S*. Typhimurium strain and its isogenic ∆*yobH* mutant in HeLa cells and RAW264.7 mouse macrophages. Additionally, we constructed a complemented ∆*yobH* mutant (∆*yobH* + *yobH-FLAG-kan*), by inserting *yobH* into the chromosome of the ∆*yobH* mutant as described in Fig. S1, which was also assessed in the invasion assays. Furthermore, the ∆*hilD* and ∆*ssrB* mutants were also tested in these assays as positive and negative controls, respectively; SsrB is a transcriptional regulator that is required for *Salmonella* intracellular replication but not for invasion of host cells^2,40^. The ∆*yobH* mutant showed a ∼3-fold decrease in the invasion of both HeLa cells and RAW264.7 macrophages with respect to the WT and the complemented ∆*yobH* + *yobH-FLAG-kan* strains (Fig. 2A,B). As expected, the ∆*hilD* mutant was unable to invade the HeLa cells and RAW264.7 macrophages, whereas the ∆*ssrB* mutant invaded these cells at similar levels to those showed by the WT and the complemented ∆*yobH* + *yobH-FLAG-kan* strains (Fig. 2A,B). Important to note, the number of bacteria present in the starting inoculums used in the invasion assays showed a variation of only 18% between the different strains tested (Fig. S2). Together, these results indicate that YobH is a novel invasion factor of *S*. Typhimurium.

### HilD controls expression of *yobH* through SprB

We sought to determine if HilD regulates the *yobH* virulence gene directly or indirectly. For this, we performed electrophoretic mobility shift assays (EMSAs) by using affinity-purified maltose-binding protein (MBP)-HilD and a DNA fragment spanning the intergenic region upstream of *yobH*. DNA fragments carrying the regulatory region of *hilA* or *sopB* were also assessed in these assays as positive and negative controls, respectively; HilD binds to *hilA* but not to *sopB*^25^. As show in Fig. S3A, MBP-HilD did not shift the *yobH* fragment or that of *sopB*, even at the highest protein concentration tested (1 µM). In contrast, MBP-HilD shifted the positive control, *hilA*, at concentrations from 0.1 to 1 µM (Fig. S3B). These assays indicate that HilD does not interact with the regulatory region of *yobH*; alternatively, HilD could require an additional factor to bind to *yobH*.

To investigate whether HilD requires another *S*. Typhimurium regulator to induce the expression of *yobH*, we monitored the activity of the *yobH-cat* fusion in the WT *E. coli* MC4100 strain, which lacks HilD and the other *Salmonella*-specific regulators, in the presence of the pK6-HilD plasmid expressing HilD or in the presence of the pMPM-K6Ω vector. As a positive control, activity of the *hilA-cat* fusion was also tested; *hilA* is directly regulated by HilD^47,48^. As expected, the *yobH-cat* and *hilA-cat* fusions showed low or undetectable expression levels in *E. coli* (Fig. S3C,D). The activity of *hilA-cat*, but not that of *yobH-cat*, was induced by HilD in *E. coli* (Fig. S3C,D), indicating that an additional factor, present in *S*. Typhimurium SL1344 but not in *E. coli* MC4100, is required for the HilD-mediated expression of *yobH*.

**Figure 3:**
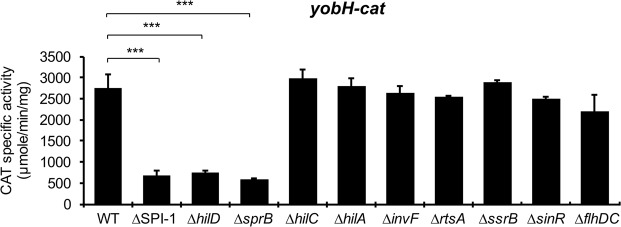
SprB is required for the expression of *yobH* in LB. Activity of the *yobH-cat* transcriptional fusion from the pyobH-cat plasmid, was determined in the WT *S*. Typhimurium SL1344 strain and its isogenic ∆SPI-1, ∆*hilD*, ∆*sprB*, ∆*hilC*, ∆*hilA*, ∆*invF*, ∆*rtsA*, ∆*ssrB*, ∆*sinR* and ∆*flhDC* mutants. CAT specific activity was quantified from samples of bacterial cultures grown for 9 h in LB at 37 °C. Means and standard deviations from three independent experiments performed in duplicate are shown. Statistically different values are indicated (***p < 0.001).

Several studies have shown that HilD induces expression of a high number of virulence genes through distinct regulatory cascades involving the HilA, InvF, HilC, RtsA, SsrA/SsrB and FlhDC transcriptional regulators^2,25,28,29,32,33^. To determine if any of these regulators are required for the HilD-mediated expression of *yobH*, activity of the *yobH-cat* fusion was measured in the WT *S*. Typhimurium strain and its isogenic ∆*hilA*, ∆*invF*, ∆*hilC*, ∆*rtsA*, ∆*ssrB* and ∆*flhDC* mutants. As positive controls, the ∆SPI-1 and ∆*hilD* mutants were also tested; SPI-1 encodes HilD, HilA, InvF and HilC^2^. Activity of the *yobH-cat* fusion was affected in the ∆SPI-1 and ∆*hilD* mutants, but not in the ∆*hilA*, ∆*invF*, ∆*hilC*, ∆*rtsA*, ∆*ssrB* and ∆*flhDC* mutants, with respect to the WT strain (Fig. 3), suggesting that HilD induces expression of *yobH* through a regulatory cascade different to those well characterized before this study.

**Figure 4:**
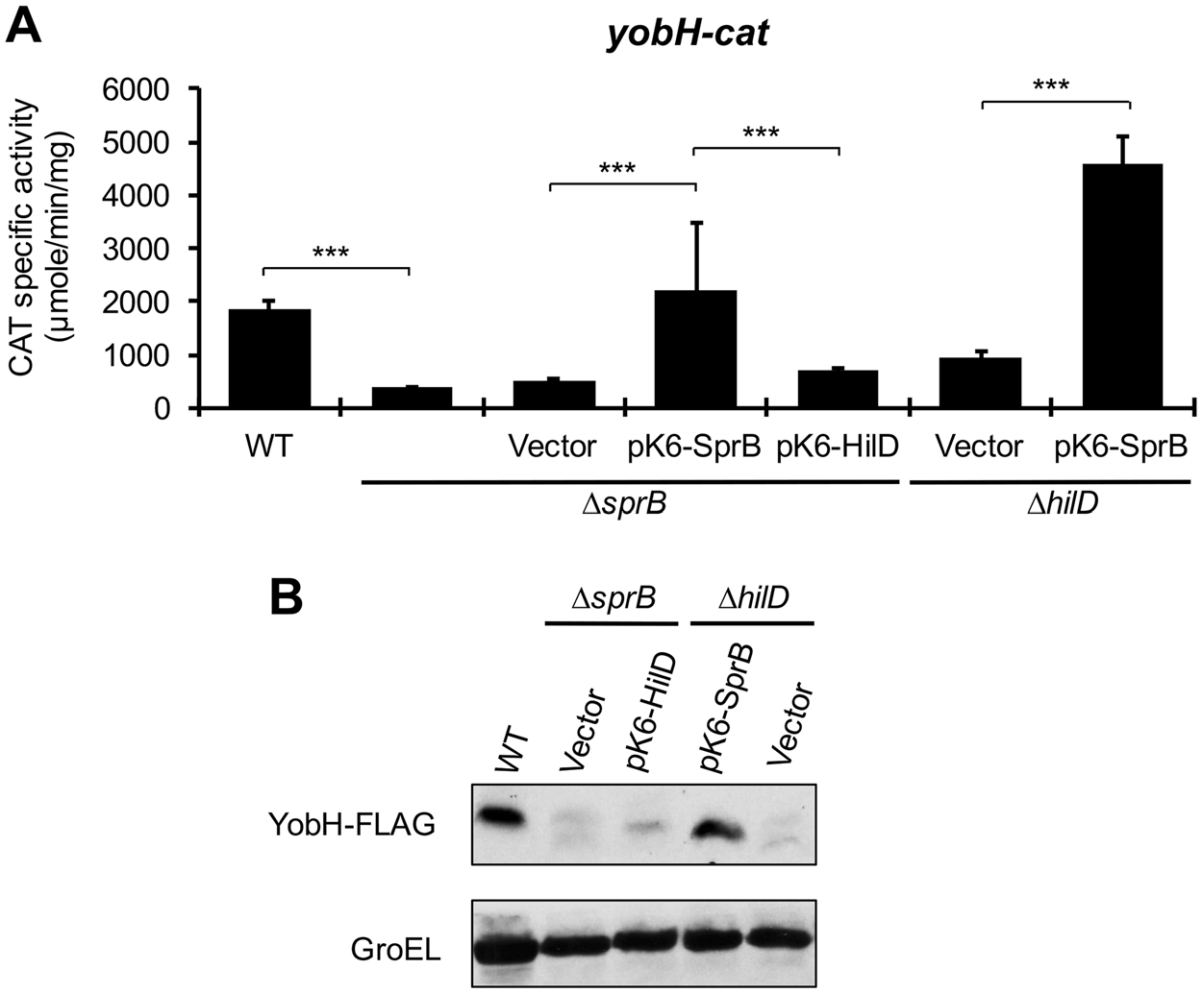
HilD induces the expression of *yobH* through SprB. (**A**) Activity of the *yobH-cat* transcriptional fusion from the pyobH-cat plasmid, was determined in the WT *S*. Typhimurium SL1344 strain and its isogenic ∆*sprB* mutant containing or not the pMPM-K6Ω vector, or the pK6-SprB or pK6-HilD plasmids, as well as in the ∆*hilD* mutant containing the pMPM-K6Ω vector or the pK6-SprB plasmid. Means and standard deviations from three independent experiments performed in duplicate are shown. Statistically different values are indicated (***p < 0.001). (**B**) Expression of YobH-FLAG in WT *S*. Typhimurium SL1344 strain and its isogenic ∆*sprB* mutant containing the pMPM-K6Ω vector or the pK6-HilD plasmid, as well as in the ∆*hilD* mutant containing the pMPM-K6Ω vector or the pK6-SprB plasmid, was analyzed by Western blotting by using an anti-FLAG monoclonal antibody. GroEL was detected as a loading control with an anti-GroEL polyclonal antibody. Blots were cropped from different parts of the same gel. CAT specific activity and YobH-FLAG expression were quantified from samples of bacterial cultures grown for 9 h in LB at 37 °C.

Previous RNA-seq analyses indicate that HilD induces expression of two additional *Salmonella*-specific transcriptional regulators, SprB and SinR^28,45^, encoded in SPI-1 and SPI-6, respectively. Moreover, we previously confirmed that HilD directly induces expression of *sinR*^39^. SprB has been involved in the expression of several *S*. Typhimurium virulence genes^28,46^, whereas SinR remains uncharacterized. To investigate whether SprB and/or SinR are involved in the HilD-mediated expression of *yobH*, we monitored the activity of the *yobH-cat* fusion in the ∆*sprB* and ∆*sinR* mutants. Surprisingly, the activity of this fusion was reduced in the ∆*sprB* mutant, as in the ∆SPI-1 and ∆*hilD* mutants, whereas it was not affected in the ∆*sinR* mutant (Fig. 3), suggesting that SprB is required for the expression of *yobH*. Expression of SprB from the pK6-SprB plasmid, under an arabinose inducible promoter, restored the activity of *yobH-cat* in both the ∆*sprB* and the ∆*hilD* mutants (Fig. 4A). In contrast, expression of HilD from the pK6-HilD plasmid induced the activity of *yobH-cat* in the ∆*hilD* mutant (Fig. 1A), but not in the ∆*sprB* mutant (Fig. 4A). Similarly, SprB restored the expression of YobH-FLAG in the ∆*hilD* mutant, whereas HilD was unable to induce the expression of YobH-FLAG in the ∆*sprB* mutant (Fig. 4B). Altogether, these results support that SprB acts downstream of HilD for the expression of *yobH*.

**Figure 5:**
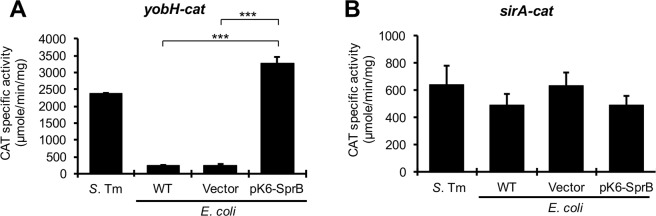
SprB induces expression of *yobH* in the absence of *Salmonella*-specific regulators. Activity of the *yobH-cat* (**A**) and *sirA-cat* (**B**) transcriptional fusions from the pyobH-cat and psirA-cat plasmids, respectively, was determined in the WT *S*. Typhimurium SL1344 strain and in the WT *E. coli* MC4100 strain containing or not the pMPM-K6Ω vector or the pK6-SprB plasmid expressing SprB under an arabinose inducible promoter. CAT specific activity was quantified from samples of bacterial cultures grown for 9 h in LB containing 0.001% L-arabinose, at 37 °C. Means and standard deviations from three independent experiments performed in duplicate are shown. Statistically different values are indicated (***p < 0.001).

Results from a chromatin immunoprecipitation sequencing (ChIP-seq) analysis revealed that SprB binds to the regulatory region of *yobH in vivo*^28^. We sought to confirm the SprB binding on *yobH* by EMSAs; however, we were unable to purify the 6XHis-tagged SprB protein, probably due to its high insolubility. Alternatively, we investigated if SprB requires any other *Salmonella*-specific regulator to induce the expression of *yobH*. For this, the activity of the *yobH-cat* fusion was tested in the WT *S*. Typhimurium strain and in the WT *E. coli* MC4100 strain carrying the pMPM-K6Ω vector or the pK6-SprB plasmid. Activity of a *cat* transcriptional fusion of *sirA*, a gene expected to be not controlled by SprB, was also tested as negative control; an ortholog of *sirA* (*uvrY*) is present in *E. coli* K-12^49,50^. The presence of SprB induced the activity of *yobH-cat* in *E. coli* to levels similar to those reached by this fusion in the WT *S*. Typhimurium strain (Fig. 5A); in contrast, SprB did not affect the activity of the *sirA-cat* fusion (Fig. 5B). These results are in line with the notion that SprB directly activates expression of *yobH*.

**Figure 6:**
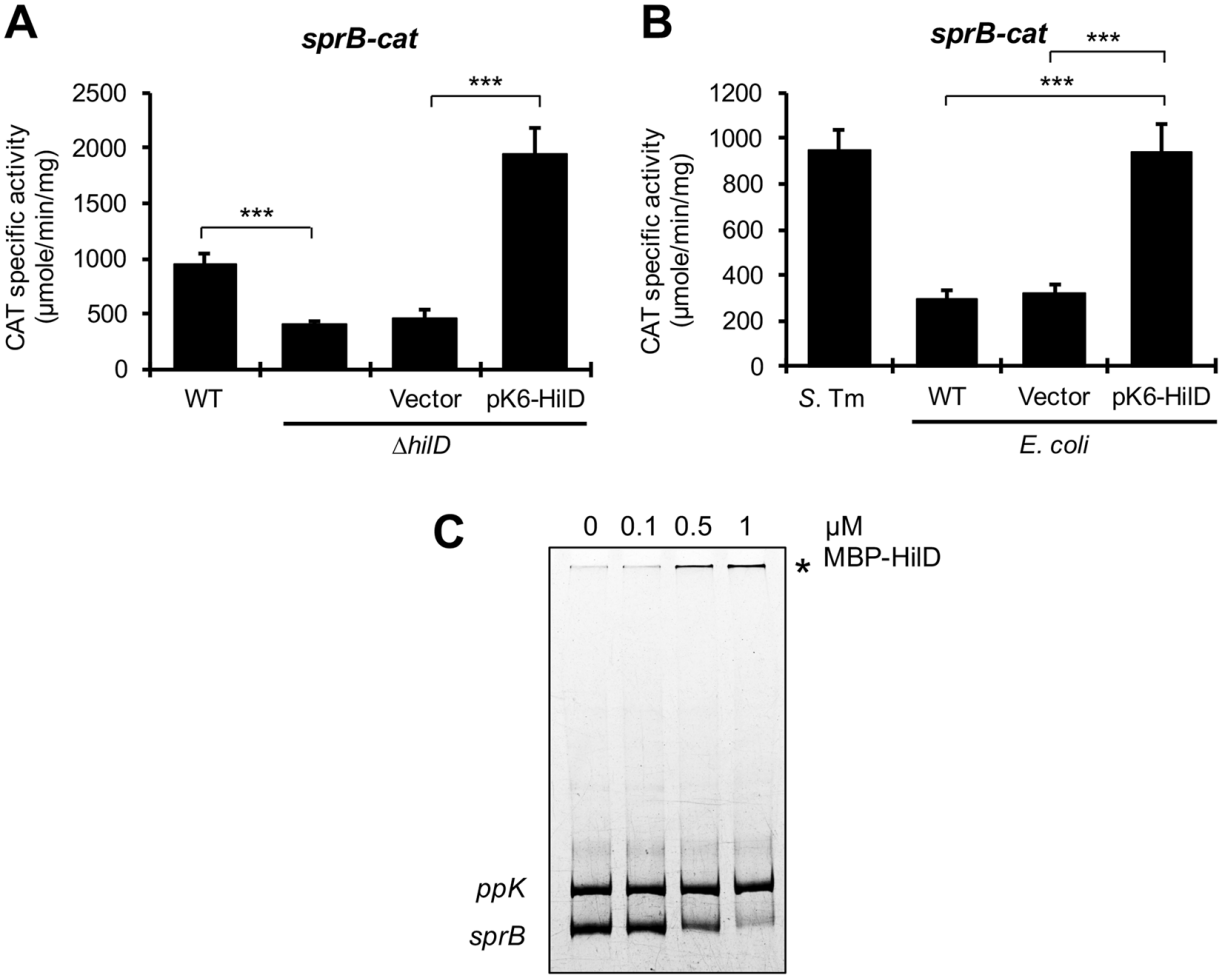
HilD positively controls expression of *sprB*. Activity of the *sprB-cat* transcriptional fusion from the psprB-cat plasmid, was determined in the WT *S*. Typhimurium SL1344 strain (**A,B**) and its isogenic ∆*hilD* mutant containing or not the pMPM-K6Ω vector or the pK6-HilD plasmid (**A**), as well as in the WT *E. coli* MC4100 strain containing or not the pMPM-K6Ω vector or the pK6-HilD plasmid (**B**). CAT specific activity was quantified from samples of bacterial cultures grown for 9 h in LB at 37 °C. Means and standard deviations from three independent experiments performed in duplicate are shown. Statistically different values are indicated (***p < 0.001). EMSAs were performed with purified MBP-HilD (0, 0.1, 0.5 and 1 µM) and a DNA fragment containing the regulatory region of *sprB* (**C**). A DNA fragment containing the regulatory region of *ppK* was used as a negative internal control. The DNA-protein complexes, indicated by an asterisk, were resolved in a nondenaturing 6% polyacrylamide gel and stained with ethidium bromide.

The results described above strongly suggest that HilD positively regulates the expression of *sprB*. To confirm this, we quantified the expression of *sprB* in the WT *S*. Typhimurium strain and its isogenic ∆*hilD* mutant. Expression of *sprB* seems to be controlled by both the regulatory region upstream of *hilC*, which generates a *hilC*-*sprB* transcript, and that located upstream of *sprB*^28^. HilD regulation on the promoter upstream of *hilC* has been extensively shown in previous studies^28,30,38,48,51^. Thus, we evaluated the effect of HilD on the regulatory region upstream of *sprB* by constructing and analyzing a *sprB-cat* transcriptional fusion carrying this region. As shown in Fig. 6A, activity of the *sprB-cat* fusion was 2-fold reduced in the ∆*hilD* mutant, compared with its activity in the WT strain; furthermore, expression of HilD from the pK6-HilD plasmid increased 3-fold the activity of this fusion in the ∆*hilD* mutant, indicating that HilD induces expression of *sprB* by also acting on the regulatory region upstream of this gene.

To investigate whether HilD regulates expression of *sprB* (by acting on the regulatory region upstream of this gene) directly or through an additional *Salmonella*-specific factor, we determined the activity of the *sprB-cat* fusion in the WT *E. coli* MC4100 strain carrying the pK6-HilD plasmid or the pMPM-K6Ω vector. As expected, activity of the *sprB-cat* fusion was 3-fold lower in the *E. coli* strain than in the WT *S*. Typhimurium strain (Fig. 6B). Expression of HilD from pK6-HilD induced 3-fold the activity of *sprB-cat* in the *E. coli* strain (Fig. 6B), supporting that HilD induces expression of *sprB* directly. In agreement to these results, EMSAs revealed that purified MBP-HilD binds to the regulatory region upstream of *sprB*, from a concentration of 0.5 µM, but it does not bind to the regulatory region upstream of *ppK*, used as a negative control in these assays (Fig. 6C). Furthermore, previous results from ChIP-seq analyses indicate that HilD binds to the intergenic region upstream of *sprB in vivo*^28,38^.

Collectively, these results indicate that HilD positively regulates the expression of *yobH* through SprB.

### HilD counteracts H-NS-mediated repression on *sprB*

**Figure 7:**
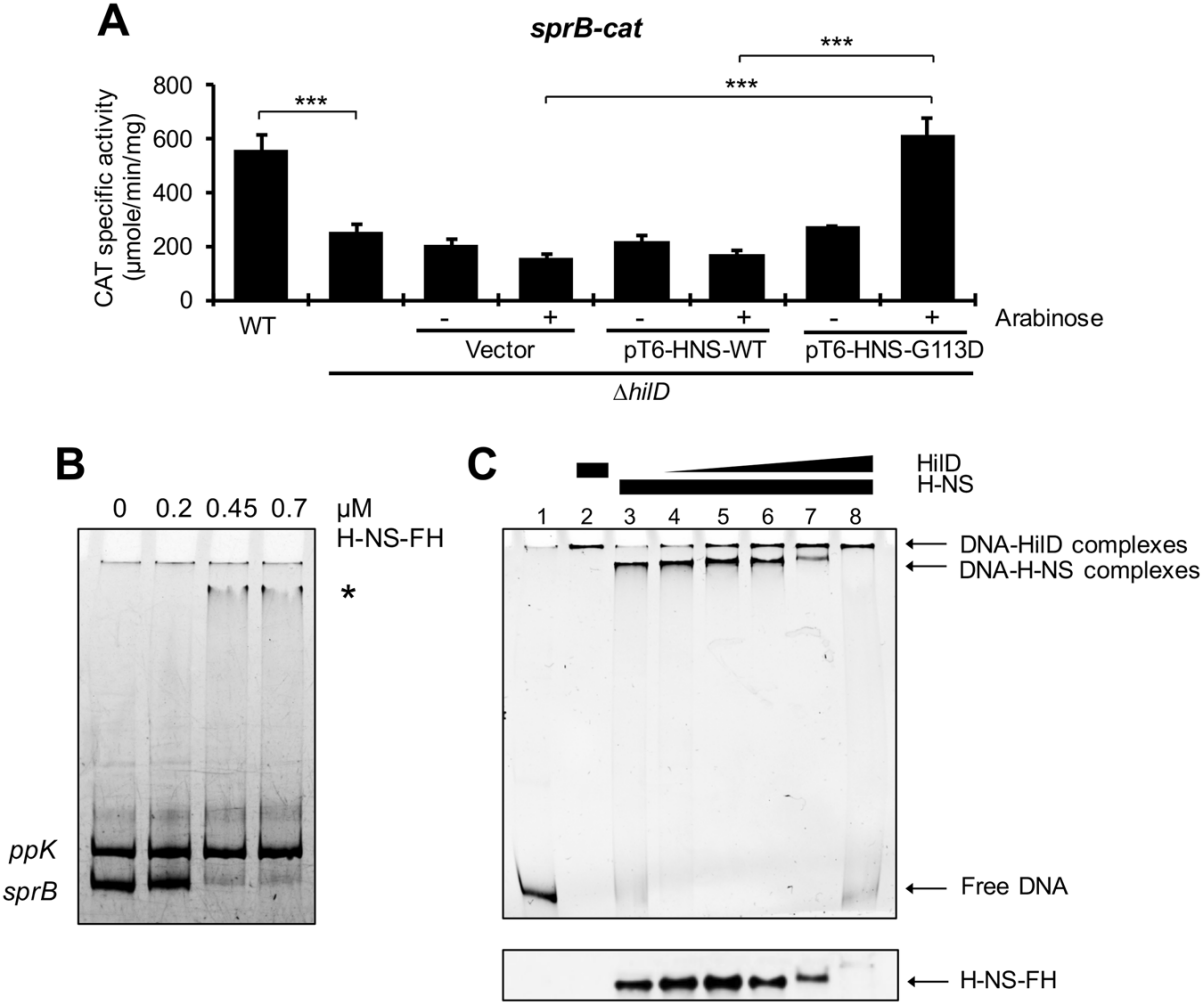
HilD directly displaces H-NS-mediated repression on *sprB*. (**A**) Activity of the *sprB-cat* transcriptional fusion from the psprB-cat plasmid, was determined in the WT *S*. Typhimurium SL1344 strain and its isogenic ∆*hilD* mutant containing or not the pMPM-T6Ω vector, or the pT6-HNS-WT or pT6-HNS-G113D plasmids, with (+) and without (−) induction (0.1% L-arabinose). CAT specific activity was quantified from samples of bacterial cultures grown for 9 h in LB at 37 °C. Means and standard deviations from three independent experiments performed in duplicate are shown. Statistically different values are indicated (***p < 0.001). (**B**) EMSAs were performed with purified H-NS-FH (0, 0.2, 0.45 and 0.7 µM) and a DNA fragment containing the regulatory region of *sprB*. A DNA fragment containing the regulatory region of *ppK* was used as a negative internal control. The DNA-protein complexes, indicated by an asterisk, were resolved in a nondenaturing 6% polyacrylamide gel and stained with ethidium bromide. (**C**) Competitive nonradioactive EMSAs between H-NS and HilD on the regulatory region of *sprB*. Purified H-NS-FH protein was added at 0.6 µM (lanes 3 to 8) and purified MBP-HilD protein was added at 0.2, 0.4, 0.6, 0.8 and 1 µM (lanes 4 to 8, respectively). No proteins were added in lane 1 and MBP-HilD was added at 1 µM in lane 2. The DNA-protein complexes were resolved in a nondenaturing 6% polyacrylamide gel. The upper panel shows the protein-DNA complexes stained with ethidium bromide and the lower panel shows the immunoblot detection of H-NS-FH from the DNA-protein complexes. Blots for DNA or protein detection were cropped from different gels.

HilD induces expression of target genes mainly by counteracting H-NS-mediated repression on the respective promoters^2,36^. To know whether HilD induces expression of *sprB* by a similar way, we analyzed if inactivation of H-NS leads to HilD-independent expression of this gene. Since a *Salmonella* ∆*hns* mutant exhibits a severe growth defect^52,53^, we inactivated H-NS activity by the overexpression of the H-NS^G113D^ dominant negative mutant, which is affected in DNA binding activity but still forms heterodimers with the WT H-NS monomers^54^. For this purpose, activity of the *sprB-cat* fusion was quantified in the WT *S*. Typhimurium strain and its isogenic ∆*hilD* mutant containing the pT6-HNS-G113D or pT6-HNS-WT plasmids, which express H-NS^G113D^ and WT H-NS, respectively, or containing the empty vector pMPM-T6Ω. Expression of H-NS^G113D^, but not WT H-NS, increased the activity of *sprB-cat* in the ∆*hilD* mutant, at similar levels to those reached by this fusion in the WT strain (Fig. 7A). Consistently, activity of *sprB-cat* was also induced in an *E. coli* ∆*hns* mutant, compared with the WT *E. coli* strain (Fig. S4). Furthermore, EMSAs revealed that purified H-NS-FLAG-His (H-NS-FH) protein binds to the regulatory region upstream of *sprB*, from a concentration of 0.45 µM, but it does not bind to the regulatory region upstream of *ppK*, used as a negative control in these assays, even at the highest protein concentration tested (0.7 µM) (Fig. 7B). Previous genome-wide binding studies indicate that H-NS interacts with the region upstream of *sprB in vivo*^52,53^. In contrast to the observed for *sprB*, the activity of the *yobH-cat* fusion was not increased in the *E. coli* ∆*hns* mutant and H-NS-FH did not bind to the regulatory region upstream of *yobH* (Fig. S5). These results show that H-NS directly represses expression of *sprB*, but not of *yobH*, and that when the activity of H-NS is inactivated, or when H-NS is absent, expression of *sprB* becomes independent of HilD, which supports that HilD acts on this gene as an anti-H-NS factor.

To determine whether HilD indeed displaces H-NS from *sprB*, we performed competitive EMSAs. A DNA fragment carrying the regulatory region of *sprB* was first incubated with a constant concentration of H-NS-FH (0.6 µM) and then increasing amounts of MBP-HilD (0.2, 0.4, 0.6, 0.8 and 1 µM) were added. Binding reactions containing only H-NS-FH or MBP-HilD were also tested. The DNA-protein complexes were detected by staining the DNA fragments with ethidium bromide; additionally, the presence of H-NS-FH on these complexes was detected by Western blot with anti-FLAG antibodies. As shown in Fig. 7C, the DNA-H-NS complex was shifted by the presence of MBP-HilD to a slower-migrating complex similar to that formed only by MBP-HilD (upper panel); furthermore, the immunoblots showed that the presence of MBP-HilD decreased the amount of H-NS-FH bound to the tested DNA fragment (lower panel), which indicates that HilD is able to remove H-NS from *sprB*.

Altogether, these results demonstrate that HilD induces expression of *sprB* by antagonizing H-NS-mediated repression on this gene.

### The HilD-SprB regulatory cascade controls expression of the *slrP* and *ugtL* virulence genes

**Figure 8:**
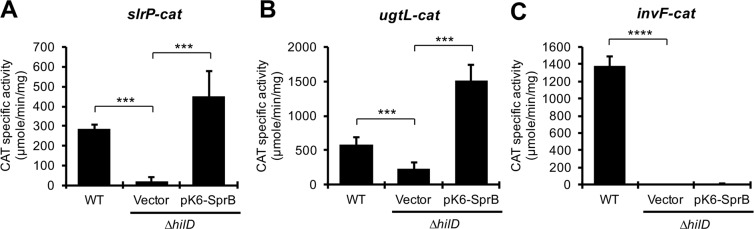
The HilD-SprB regulatory cascade induces expression of the *slrP* and *ugtL* genes. Activity of the *slrP-cat*, *ugtL-cat* and *invF-cat* transcriptional fusions from the pslrP-cat, pugtL-cat and pinvF-cat plasmids, was determined in the WT *S*. Typhimurium SL1344 strain and its isogenic ∆*hilD* mutant containing the pMPM-K6Ω vector or the pK6-SprB plasmid. CAT specific activity was quantified from samples of bacterial cultures grown for 9 h in LB at 37 °C. Means and standard deviations from three independent experiments performed in duplicate are shown. Statistically different values are indicated (***p < 0.001; ****p < 0.0001).

Previous RNA-seq analyses indicate that HilD and SprB positively controls expression of several other genes in common, in addition to *yobH*, including *slrP* and *ugtL*^28^, which have been involved in *Salmonella* virulence^55–59^. To further define if HilD and SprB also act in a cascade fashion on *slrP* and *ugtL*, we constructed and analyzed *cat* transcriptional fusions carrying the regulatory region of the *slrP* or *ugtL* genes. Activity of the *slrP-cat* and *ugtL-cat* fusions was quantified in the WT *S*. Typhimurium strain and its derivative ∆*hilD* mutant containing the pK6-SprB plasmid or the pMPM-K6Ω vector. As a negative control, an *invF-cat* transcriptional fusion was also assessed; HilD induces expression of *invF* through HilA^27,60,61^. The three fusions tested showed a decreased activity in the ∆*hilD* mutant, with respect to their activity in the WT strain (Fig. 8A–C). Expression of SprB from pK6-SprB induced activity of the *slrP-cat* and *ugtL-cat* fusions, but not that of the *invF-cat* fusion, in the ∆*hilD* mutant (Fig. 8A–C), supporting that HilD controls the expression of *slrP* and *ugtL* through SprB, which is in agreement with data from ChIP-seq analyses showing SprB binding, but not HilD binding, on the regulatory regions of *slrP* and *ugtL*^28^.

Thus, our results, together with previous studies, indicate that the regulatory cascade formed by HilD and SprB controls expression of a subset of *Salmonella* virulence genes, including *yobH*, *slrP* and *ugtL*.

## Discussion

Acquisition of SPI-1 was a pivotal event for the evolution of *Salmonella* pathogenicity, not only by the virulence factors encoded in this island, which provide ability to invade host cells, but also by the additional factors for invasion encoded outside SPI-1 that have been recruited through the control of their expression by the SPI-1 regulator HilD^2,28^.

In this study, we identify a novel invasion factor, YobH, whose expression is controlled by HilD. Our results demonstrate that YobH is required for the *S*. Typhimurium invasion of HeLa cells and mouse macrophages. In agreement with these results, a previous analysis by transposon-directed insertion-site sequencing (TraDIS) supports that YobH plays a role in the intestinal colonization of *S*. Typhimurium in chicks and cows, but not in the systemic infection in the mouse model^56^. The *yobH* gene is located outside SPI-1, in a chromosomal region conserved in many bacteria, including *S. bongori* and *E. coli* K-12; YobH shares 79% sequence identity with its ortholog from *E. coli* K-12. A previous study indicates that HilD directly regulates expression of the *flhDC* operon, encoding the master regulator of the flagellar genes, which is also conserved in *E. coli* K-12 and many other bacteria^32,33^. YobH and its orthologs from different bacteria are on average 80 amino acids long and have no an assigned function; they are annotated as putative membrane, exported or uncharacterized proteins. Our preliminary results support that YobH is secreted in *S*. Typhimurium (data not shown). How is YobH secreted and what is the specific function of YobH for invasion, are topics of our current investigation.

HilD induces expression of a high number of target genes by acting directly or through distinct regulators, in growth conditions that somehow resemble the intestinal environment (SPI-1-inducing conditions), such as those that we assessed in this study^2^. At present, three different regulatory cascades headed by HilD have been well characterized: the HilD-HilA-InvF, HilD-SsrA/SsrB and HilD-FlhDC cascades^2,25,27,32,33,36,37^. Additionally, HilD forms a feed-forward positive loop with HilC and RtsA, which amplifies the activation of the HilD-HilA-InvF cascade^30,31^, and probably also the activation of the other regulatory cascades and genes controlled directly by HilD. Our data, together with previous results obtained from genome-wide expression and binding analyses^28^, define an additional cascade formed by HilD to induce expression of virulence genes. In this regulatory cascade, HilD induces expression of the *yobH, slrP* and *ugtL* virulence genes through SprB, a *Salmonella*-specific LuxR-like regulator encoded in SPI-1. Previous studies revealed that HilD and RtsA induce expression of *slrP* by an undefined way^62,63^. SlrP (*Salmonella* leucine-rich repeat protein) is an effector protein with ubiquitin ligase activity that is translocated into mammalian cells through both T3SS-1 and T3SS-2^63,64^. Previous reports support that SlrP plays a role in the intestinal colonization of *S*. Typhimurium in chicks, pigs, cows and mice, but not in the systemic infection in the mouse model^55,56^. UgtL is an inner membrane protein that mediates resistance to antimicrobial peptides by modifying lipid A in the lipopolysaccharide^57,59^; furthermore, it is involved in the activation of the PhoP/PhoQ two-component regulatory system in response to mildly acidic pH^58^. UgtL is required by *S*. Typhimurium for killing^58^ and for the intestinal colonization of mice^59^; moreover, TraDIS analysis supports that UgtL is important for the intestinal colonization of *S*. Typhimurium in pigs^56^. Importantly to note, expression of both *slrP* and *ugtL* is also controlled directly by the PhoP/PhoQ two-component system, in growth conditions that somehow mimic the intracellular environment of host cells (SPI-2-inducing conditions)^63,65^, where the HilD-mediated regulation on target genes is not evident^25,36,63^; in contrast, expression of *yobH* seems to be not regulated by PhoP/PhoQ^45^. PhoP forms with the SlyA regulator a feed-forward loop that controls expression of *ugtL* in SPI-2-inducing conditions^65,66^. Thus, expression of both *ugtL* and *slrP* is controlled by at least two distinct regulatory mechanisms that act in response to different environmental conditions. HilD-SprB and PhoP-SlyA would induce expression of *ugtL* and *slrP* in different niches where the activity of these genes is required for the *Salmonella* infection of hosts. For instance, activity of UgtL is needed for the intestinal colonization and for the systemic infection of mice^58,59^. On another hand, it is tempting to speculate that HilD-SprB helps to reach the levels of UgtL required for the subsequent UgtL-mediated activation of the PhoP/PhoQ system in response to acidic pH^58^, a cue present in the intracellular environment. Following this idea, it has been shown that activated PhoP represses expression of *hilD*, *hilA* and *rtsA*, and thus the SP-1 invasion genes^67^; therefore, the HilD-SprB-UgtL-PhoP/PhoQ pathway could work as an additional negative feedback control in the complex and dynamic regulatory network governing expression of *Salmonella* invasion genes.

Global expression and binding analyses indicate that SprB positively controls expression of *yobH*, *slrP*, *ugtL* and 20 genes more^28^, all these genes located outside SPI-1, including the *sifB*, *yhgE*, *yibP*, *SL1344_3112*, *SL1344_0336* and *SL1344_0337* genes that have been associated to virulence^56,68^. Therefore, the HilD-SprB cascade represents an additional branch that further expands the HilD virulence regulon, connecting the activity of several genes located outside SPI-1 with the capability for invasion of host cells encoded within SPI-1.

Our results demonstrate that HilD positively controls expression of *sprB* by acting on the regulatory region upstream of this gene. Previous studies indicate that HilD can also control expression of *sprB* by acting on the *hilC* gene, located upstream of *sprB*; a *hilC-sprB* transcript was detected in a previous study^28^ and direct regulation of *hilC* by HilD is well documented^28,30,38,48,51^. We show that HilD induces expression of *sprB* by directly displacing the repressor H-NS from the regulatory region upstream of this gene; a mechanism that HilD follows to induce expression of other target genes^36,37,41–43^. H-NS represses expression of *hilC*^43,53,69,70^, which suggest that HilD induces expression of the *hilC-sprB* transcript also by antagonizing H-NS mediated repression. In contrast to HilD, which is required for the expression of target genes only in the presence of H-NS, we found that SprB is required for the expression of *yobH* even in the absence of H-NS, which supports that it does not act as an anti-H-NS factor. There is growing evidence to suggest that other LuxR-like regulators mainly act as classical activators, which induce expression of target genes by favoring binding of the RNA polymerase on promoters^71–74^. Whether SprB antagonizes a repressor different to H-NS or whether it acts as a classical activator remains to be elucidated.

**Figure 9:**
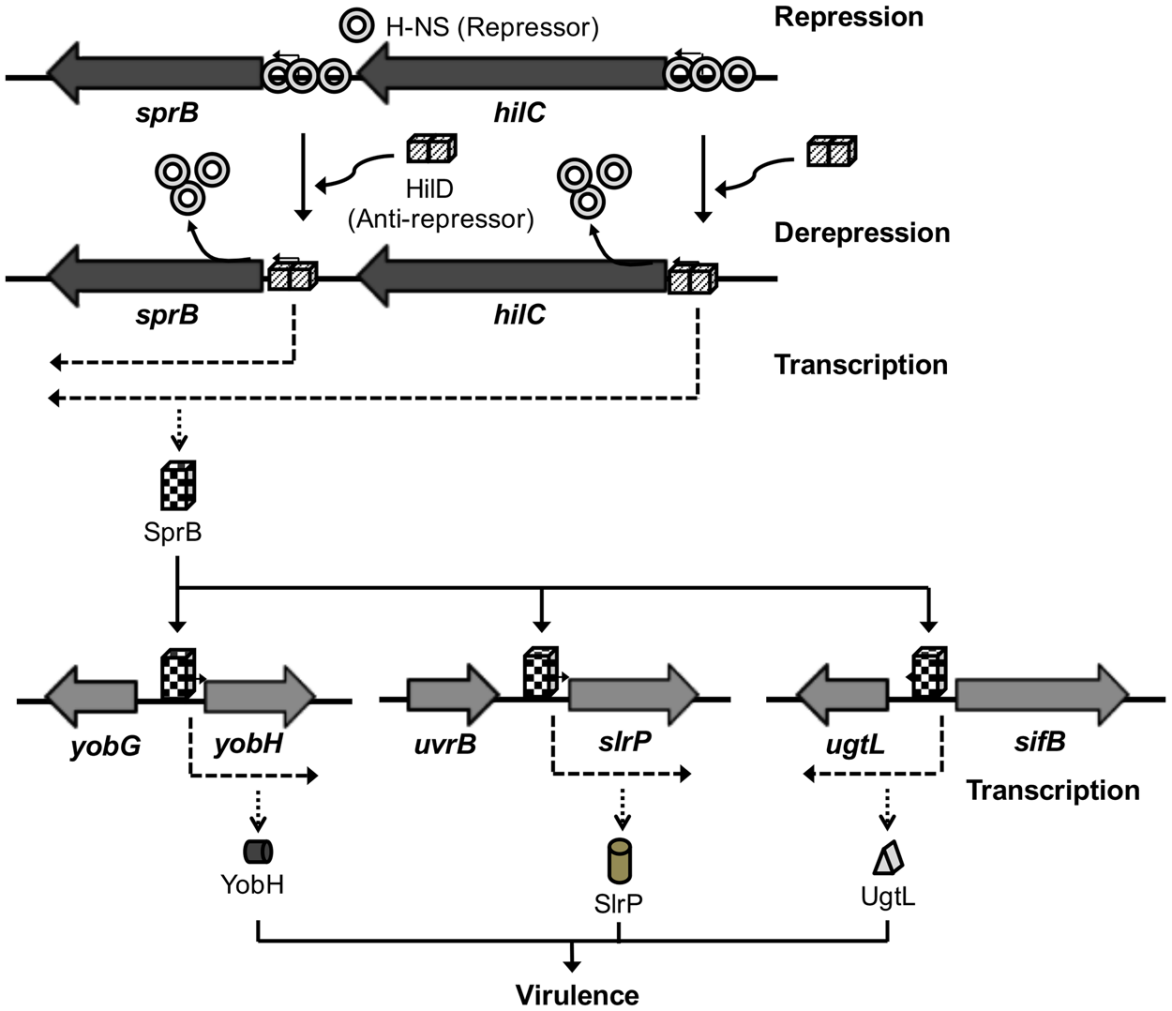
Model for the expression of YobH, SlrP and UgtL mediated by the HilD-SprB regulatory cascade. H-NS represses expression of *sprB* by binding the two promoter regions transcribing this gene. HilD binds to and thus displaces the H-NS repressor complex from these promoter regions, which allows expression of SprB that finally activates transcription of the *yobH*, *slrP* and *ugtL* virulence genes. Transcription of *sprB* from the promoter upstream of *hilC* and the effect of HilD and H-NS on this promoter were reported previously^28,30,43,48,69^. The previously defined regulation of *ugtL* and *slrP* by SlyA and/or PhoP is not depicted in the model but it is described in text.

Our data reveal a novel *Salmonella* invasion factor and further define an additional regulatory cascade mediated by HilD for the expression of *Salmonella* virulence genes. A model that summarizes the results from this study is depicted in Fig. 9.

## Methods

### Bacterial strains and growth conditions

Bacterial strains used in this study are listed in Table S1. Bacterial cultures for the determination of chloramphenicol acetyl transferase (CAT) activity and for Western blot assays were grown in LB as described previously^25,26,36^. When necessary, the medium was supplemented with the following antibiotics: ampicillin (200 μg/ml), streptomycin (100 μg/ml), kanamycin (20 μg/ml) or tetracycline, (12 μg/ml).

### Construction of plasmids

Plasmids and primers used in this study are listed in Tables S1 and S2, respectively. To generate the *yobH-cat*, *sprB-cat*, *slrP-cat* and *ugtL-cat* transcriptional fusions, the regulatory regions of *yobH*, *sprB*, *slrP* and *ugtL* were amplified by PCR using the primer pairs SL1770-FW22/SL1770-RV11, sprB-catF/sprB-catR, slrPB2-Fw22/slrPH3-Rv11 and ugtL-Fw/ugtL-Rv, respectively, and chromosomal DNA from the WT *S*. Typhimurium strain as template. The resulting PCR products were purified with the Zymoclean Gel DNA Recovery Kit (Zymo Research), digested with BamHI and HindIII enzymes and then cloned into the pKK232-8 vector^75^ digested with the same restriction enzymes. To construct the p2795-YobH-FLAG plasmid, the *yobH::3XFLAG* gene was amplified by PCR using the primer pair SL1770-FW22/1770-SalIRv and chromosomal DNA from the DTM128 strain as template. This PCR product was digested with SalI and BamHI enzymes and then cloned into the p2795 vector^76^ digested with the same restriction enzymes. To construct the pK6-SprB plasmid, the *sprB* structural gene was amplified by PCR using the primer pair sprB-K6NcoI/sprB-K6PstI and chromosomal DNA from the WT *S*. Typhimurium strain as template. This PCR product was digested with NcoI and PstI enzymes and then cloned into the pMPM-K6Ω vector^77^ digested with the same restriction enzymes. pK6-SprB expresses SprB from an arabinose-inducible promoter.

### Construction of deletion and *3XFLAG*-tagged *S*. Typhimurium mutant strains

Non-polar deletion of the *sprB*, *sinR* or *yobH* genes in the *S*. Typhimurium SL1344 strain was performed by the λRed recombinase system, as reported previously^78^, using the respective primers described in Table S2, thus generating the strains DTM121, DTM123 and DTM124, respectively. The chromosomal *yobH* gene was *3XFLAG*-tagged in the *S*. Typhimurium SL1344 strain using a previously reported method based on the λRed recombinase system^79^, thus generating the DTM127 (*yobH::3XFLAG-kan*) strain. P22 transduction was used to transfer the *yobH::3XFLAG-kan* allele from the strain DTM127 into the strains JPTM25 and DTM122, generating the strains DTM129 and DTM131, respectively. The kanamycin resistance cassette was excised from the strains DTM121, DTM124, DTM127, DTM129 and DTM131, by using the pCP20 plasmid expressing the FLP recombinase, as described previously^78^, generating the strains DTM122, DTM125, DTM128, DTM130 and DTM132, respectively. The complemented DTM126 strain was generated by inserting the *yobH::3XFLAG-kan* into the chromosome of the DTM125 strain, using a previously reported method based on the λRed recombinase system^76^ and the p2795-YobH-FLAG plasmid. All modified strains were verified by PCR amplification and sequencing.

### Chloramphenicol acetyltransferase (CAT) assays

The CAT activity and protein quantification to calculate CAT specific activities were determined as previously described^80^.

### Statistical analysis

Data were analyzed with GraphPad Prism 5.0 software (GraphPad Inc., San Diego, CA) using One-Way analysis of variance (ANOVA) with the Tukey’s multiple comparison test. A *P-*value of <0.05 was considered significant.

### Electrophoretic mobility shift assays (EMSAs)

Fragments spanning the regulatory regions of *yobH*, *hilA*, *sopB*, *sprB* and *ppK* were obtained by PCR amplification using the primer pairs SL1770-FW22/SL1770-RV11, hilA1FBamHI/hilA2RHindIII, SigDBH1F/SigDH3R, sprB-catF/sprB-catR and PPK-Fw1/PPK-Rv1, respectively, and chromosomal DNA from the WT *S*. Typhimurium strain as template. Binding reactions were performed as described previously^25,40^. For competitive EMSAs, the *sprB* fragment was first incubated with 0.6 µM H-NS–FH for 15 min and then incubated with increasing concentrations MBP-HilD for an additional 20 min. Binding mixtures were electrophoretically separated in 6% nondenaturing acrylamide in 0.5X Tris-borate-EDTA buffer, at room temperature. DNA bands were visualized by staining with ethidium bromide, in an Alpha-Imager UV transilluminator (Alpha Innotech Corp.).

### Expression and purification of proteins

Expression and purification of MBP-HilD and H-NS-FH were performed as described previously^25,40^.

### Western blotting

Western blot assays were performed as described previously^26,36^. Anti-FLAG M2 monoclonal antibodies (Sigma) were used at 1:2,000 or 1:3,000 dilutions, for detection of YobH-FLAG and H-NS-FH, respectively. Anti-GroEL polyclonal antibodies were used at a dilution of 1:100,000. Horseradish peroxidase-conjugated secondary antibodies (Pierce), anti-mouse or anti-rabbit, were used at a dilution of 1:10,000. Blots were developed by incubation with the Western Lightning Chemiluminescence Reagent Plus (Perkin-Elmer) and then exposition to KodaK X-Omat films.

### Invasion assays

Invasion of HeLa cells or RAW264.7 macrophages was determined by gentamicin protection assays as described previously^40,81^. Briefly, HeLa cells or RAW264.7 macrophages were grown in high-glucose Dulbecco’s Modified Eagle Medium (GIBCO 12100-046) supplemented with 10 mM sodium pyruvate solution, 20 mM L-glutamine and 10% (v/v) heat-inactivated fetal bovine serum, at 37 °C and a 5% CO_2_ atmosphere, in 24-well tissue culture plates. Monolayers of HeLa cells or RAW264.7 macrophages, from each well, were infected during 10 min with the respective bacterial suspension obtained from LB cultures, using a multiplicity of infection (MOI) of 40:1 and 10:1 (bacterial to eukaryotic cells), respectively. After the time of infection, monolayers were washed and then incubated during 1 h in DMEM containing 50 μg/ml gentamicin to eliminate extracellular bacteria. DMEM was removed and the HeLa cells and RAW264.7 macrophages from each well were lysed in 1 ml and 200 μl of 0.2% (w/v) sodium deoxycholate in 1X PBS, respectively. To obtain the intracellular CFUs per well, serial dilutions of each cell lysate were plated onto LB agar supplemented with 100 μg/ml streptomycin. CFUs from the starting inoculums were also quantified.

## Supplementary information

Supplementary Information

## Data Availability

All data generated or analyzed during the current study are available from the corresponding author on reasonable request.
