# Supplementary material for: HilD induces expression of a novel Salmonella Typhimurium invasion factor, YobH, through a regulatory cascade involving SprB
Source: Sci Rep. 2019 Sep 4;9:12725. doi: 10.1038/s41598-019-49192-z (PMC6726612; doi:10.1038/s41598-019-49192-z)
Supplement: Supplementary file 1 — Supplementary Information [file 41598_2019_49192_MOESM1_ESM.pdf]

Supplementary Information

HilD induces expression of a novel *Salmonella* Typhimurium invasion factor, YobH, through a regulatory cascade involving SprB

María M. Banda, Rubiceli Manzo & Víctor H. Bustamante

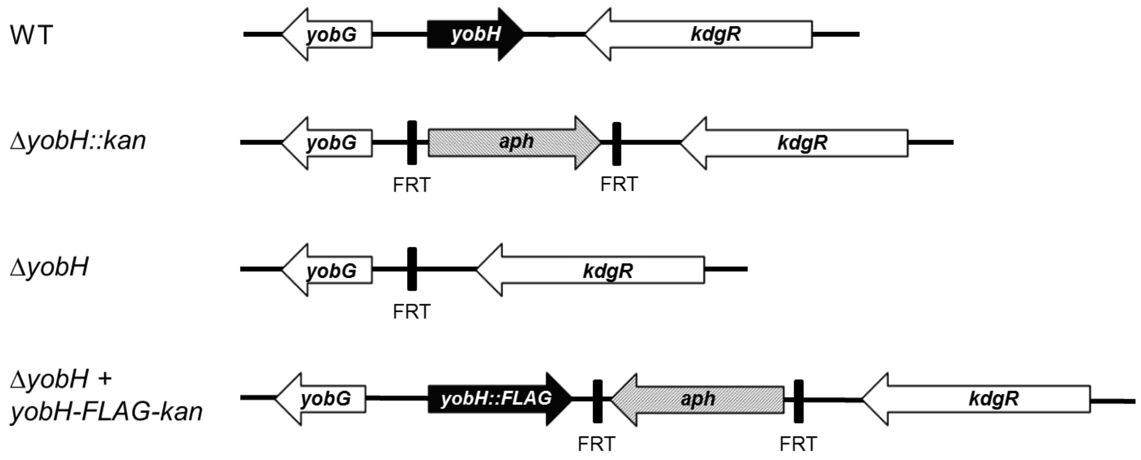

Figure S1. Generation of the Δ*yobH* mutant strains and the complemented Δ*yobH* + *yobH-FLAG-kan* strain. The Δ*yobH* mutant was generated by replacing *yobH* in the WT *S*. Typhimurium strain with a kanamycin resistance gene, using the λRed recombinase; then, the kanamycin resistance gene was excised from the Δ*yobH::kan* mutant, using the FLP recombinase, to obtain the Δ*yobH* mutant. The complemented Δ*yobH* + *yobH-FLAG-kan* strain was generated by inserting the *yobH-FLAG* gene, together with the kanamycin resistance gene in the opposite direction, into the chromosome of the Δ*yobH* mutant, using the λRed recombinase.

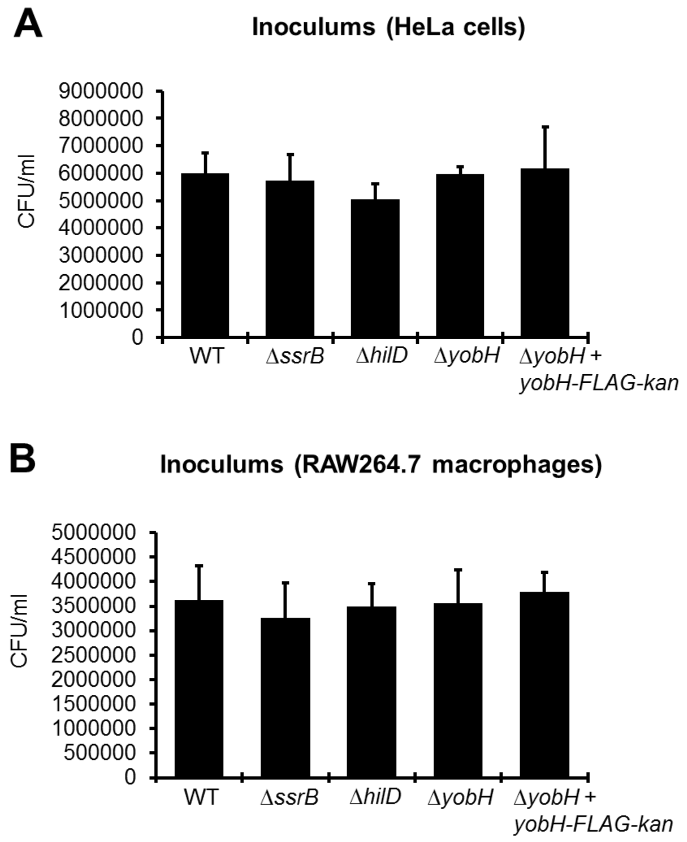

Figure S2. CFUs from the starting inoculums for the invasion assays. CFUs from the bacterial inoculums of the different strains used to infect HeLa cells (A) and RAW 264.7 macrophages (B), see Fig. 2.

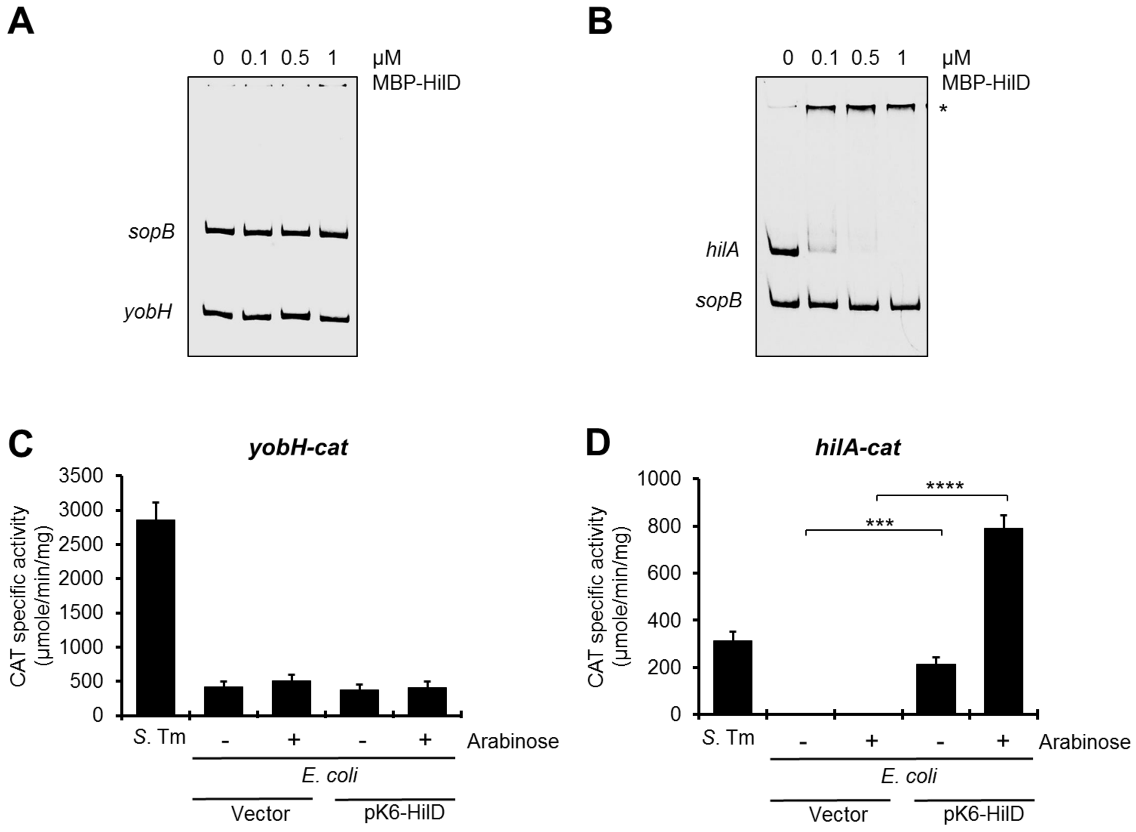

Figure S3. HilD controls expression of *yobH* indirectly. EMSAs performed with purified MBP-HilD (0, 0.1, 0.5 and 1 µM) and DNA fragments containing the regulatory region of *yobH* (A) and *hilA* (B). A DNA fragment containing the regulatory region of *sopB* was used as a negative internal control. The DNA-protein complexes, which are indicated by an asterisk, were resolved in a nondenaturing 6% polyacrylamide gel and stained with ethidium bromide. Expression of the *yobH-cat* (C) and *hilA-cat* (D) transcriptional fusions carried by the pyobH-cat and philA-cat plasmid, respectively, was evaluated in the WT *S*. Typhimurium SL1344 strain, as well as in the WT *E. coli* MC4100 strain carrying the pMPM-K6Ω vector or the pK6-HilD plasmid expressing HilD under an arabinose inducible promoter. CAT specific activity was determined from samples collected of bacterial cultures grown for 9 h at 37°C in LB containing (+) or not (-) 0.001% L-arabinose. Means and standard deviations from three independent experiments performed in duplicate are shown. Statistically different values are indicated (***, $p<0.001$; ****, $p<0.0001$).

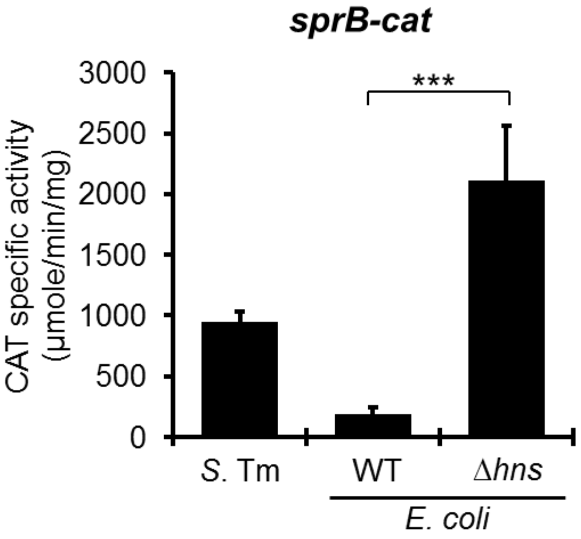

Figure S4. H-NS represses expression of *sprB*. Expression of the *sprB-cat* transcriptional fusion carried by the psprB-cat plasmid was determined in the WT *S*. Typhimurium SL1344 strain, as well as in the WT *E. coli* MC4100 strain and its isogenic Δ*hns* mutant. CAT specific activity was determined from samples collected of bacterial cultures grown for 9 h in LB at 37°C. Means and standard deviations from three independent experiments performed in duplicate are shown. Statistically different values are indicated (***, $p<0.001$).

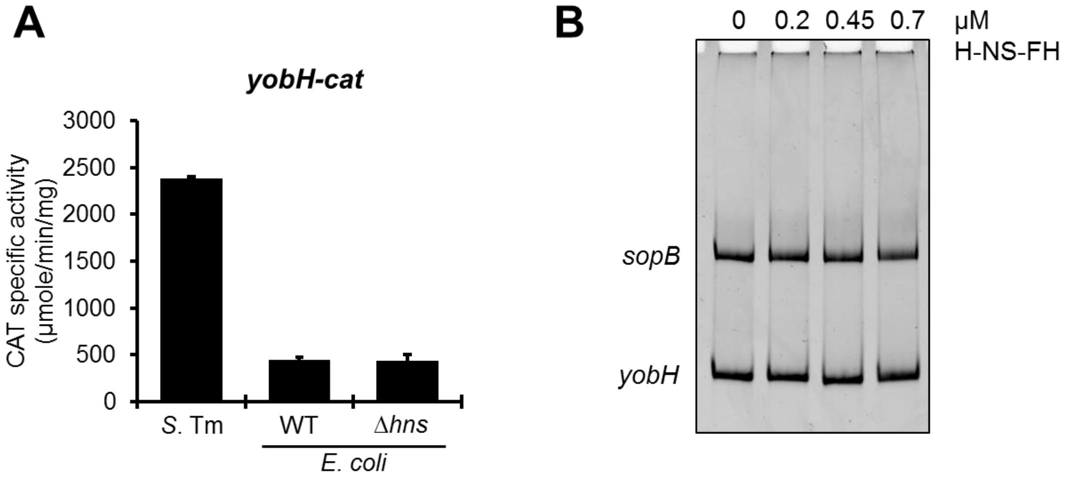

Figure S5. H-NS does not repress expression of *yobH*. (A) Expression of the *yobH-cat* transcriptional fusion carried by the pyobH-cat plasmid was determined in the WT *S*. Typhimurium SL1344 strain, as well as in the WT *E. coli* MC4100 strain and its isogenic Δ*hns* mutant. CAT specific activity was determined from samples collected of bacterial cultures grown for 9 h in LB at 37°C. Means and standard deviations from three independent experiments performed in duplicate are shown. (B) EMSA with purified H-NS-FH (0, 0.2, 0.45 and 0.7 µM) and DNA fragment containing the regulatory region of *yobH*. A DNA fragment containing the regulatory region of *sopB* was used as a negative internal control. The DNA-protein complexes were resolved in a nondenaturing 6% polyacrylamide gel and stained with ethidium bromide.

Strain or plasmid	Genotype or description	Reference or source
Strains		
S. Typhimurium		
SL1344	Wild type; *xyl*, *hisG*, *rpsL*; Sm^R	[1]
ΔSPI-1	ΔSPI-1*::kan*	[2]
VV341	Δ*hilA::kan-339*	[3]
JPTM4	Δ*invF::kan*	[4]
JPTM5	Δ*hilD::kan*	[4]
JPTM6	Δ*hilC::kan*	[4]
JPTM25	Δ*hilD*	[5]
DTM91	Δ*rtsA::kan*	[6]
DTM90	Δ*flhDC::kan*	[7]
DTM97	Δ*ssrB::kan*	[6]
DTM121	Δ*sprB::kan*	This study
DTM122	Δ*sprB*	This study
DTM123	Δ*sinR::kan*	This study
DTM124	Δ*yobH::kan*	This study
DTM125	Δ*yobH*	This study
DTM126	Δ*yobH* + *yobH::3XFLAG-kan*	This study
DTM127	*yobH::3XFLAG-kan*	This study
DTM128	*yobH::3XFLAG*	This study
DTM129	Δ*hilD yobH::3XFLAG-kan*	This study
DTM130	Δ*hilD yobH::3XFLAG*	This study
DTM131	Δ*sprB yobH::3XFLAG-kan*	This study
DTM132	Δ*sprB yobH::3XFLAG*	This study
E. coli		
DH10β	Laboratory strain	(Invitrogen)
MC4100	F^-(*araD139*) Δ(*argF-lac*)*169*λ^- e14$^-$*flhD5301* Δ(*fruK-yeiR*)725(*fruA25*) *relA1 rpsL150*(Sm^R) *rbsR22* Δ(*fimB-fimE*)*632*(::IS*1*) *deoC1*	[8]
JPMC34	MC4100 derivative, Δ*hns*	[9]
Plasmids		
pKK232-8	pBR322 derivative containing a promotorless chloramphenicol acetyltransferase (*cat*) gene, Ap^R	[10]
pyobH-cat	pKK232-8 derivative containing a *yobH-cat* transcriptional fusion from nucleotides -193 to +113[a]	This study

philA-cat	pKK232-8 derivative containing a *hilA-cat* transcriptional fusion from nucleotides -410 to +446[a]	[4]
psirA-cat	pKK232-8 derivative containing a *sirA-cat* transcriptional fusion from nucleotides -563 to +98[a]	[5]
psprB-cat	pKK232-8 derivative containing a *sprB-cat* transcriptional fusion from nucleotides -497 to +144[b]	This study
pslrP-cat	pKK232-8 derivative containing a *slrP-cat* transcriptional fusion from nucleotides -510 to +101[a]	This study
pugtL-cat	pKK232-8 derivative containing an *ugtL-cat* transcriptional fusion from nucleotides -436 to +216[a]	This study
pinvF-cat	pKK232-8 derivative containing an *invF-cat* transcriptional fusion from nucleotides -306 to +213[a]	[4]
pMPM-K6Ω	p15A derivative cloning vector containing an arabinose-inducible promoter, Kan^R	[11]
pK6-HilD	pMPM-K6Ω derivative expressing HilD under an arabinose-inducible promoter	[7]
pK6-SprB	pMPM-K6Ω derivative expressing SprB under an arabinose-inducible promoter	This study
pMPM-T6Ω	p15A derivative low-copy-number cloning vector, arabinose-inducible promoter, Tc^R	[11]
pT6-HNS-WT	pMPM-T6Ω derivative expressing WT H-NS from the arabinose-inducible promoter	[4]
pT6-HNS-G113D	pMPM-T6Ω derivative expressing H-NS^{G113D} from the arabinose-inducible promoter	[12]
pMAL-HilD1	pMAL-c2X derivative expressing MBP-HilD from a *lac* promoter, Ap^R	[4]
pBAD-H-NS-FH	pBADMycHisC derivative expressing H-NS-FH from an *ara* promoter, Ap^R	[12]
pKD46	pINT-ts derivative expressing red recombinase under an arabinose-inducible promoter, Ap^R	[13]
pKD4	pANTsγ derivative template plasmid containing the kanamycin cassette for λRed recombination, Ap^R	[13]
pSUB11	pGP704 derivative template plasmid for	

	FLAG epitope tagging	14
pCP20	Plasmid expressing FLP recombinase from a temperature-inducible promoter, Ap^R	13
p2795	pBluescript SK+ containing *aph* FRT, Ap^R Kan^R	15
p2795-YobH-FLAG	p2795 containing *yobH::3XFLAG*	This study

Table S1. Bacterial strains and plasmids used in this study. The coordinates for the *cat* fusions are indicated with respect to the primary transcriptional start site (a) or to the first base of the start codon (b), for each gene. Ap^R, ampicillin resistance; Kan^R, kanamycin resistance; Sm^R, streptomycin resistance; Tc^R, tetracycline resistance.

Primer	Sequence (5' – 3')[1, 2]	Target gene	RE[3]
**For *cat* transcriptional fusion**			
SL1770-FW22	CCTGGATCCACAGCAACAGGCATACTAC	*yobH*	BamHI
SL1770-RV11	GTTAAGCTTACTTCCCACCAGGATGCC	*yobH*	HindIII
sprB-catF	CAGAAGCTTGTCCGGCATATCGGATAGGG	*sprB*	HindIII
sprB-catR	CTGGGATCCTTTCACAAGTCGCGACGATGTG	*sprB*	BamHI
slrPB2-Fw22	TTCGGATCCAGTTGCACCAGTTACGCG	*slrP*	BamHI
slrPH3-Rv11	TCTAAGCTTAAAGGCACCTCTGTTGAGGC	*slrP*	HindIII
ugtL-Fw	CGAGGATCCGAGCGTTGAGAGATAGCACT	*ugtL*	BamHI
ugtL-Rv	CGAAAGCTTCCAGGATGCTGTCTTTTC	*ugtL*	HindIII
**For EMSAs**			
SL1770-FW22	CCTGGATCCACAGCAACAGGCATACTAC	*yobH*	BamHI
SL1770-RV11	GTTAAGCTTACTTCCCACCAGGATGCC	*yobH*	HindIII
hilA1FBamHI	ATCGGATCCCTCTGAGAACTATTTGC	*hilA*	BamHI
hilA2RHindIII	GACAAGCTTTTCTGAGCGTAGCAGGG	*hilA*	HindIII
sigDBH1F	TCCCGACAGGATCCTTTTACCC	*sopB*	BamHI
sigDH3R	CGTTGTATAAGCTTTTTTGTAG	*sopB*	HindIII
sprB-catF	CAGAAGCTTGTCCGGCATATCGGATAGGG	*sprB*	HindIII
sprB-catR	CTGGGATCCTTTCACAAGTCGCGACGATGTG	*sprB*	BamHI
PPK-Fw1	GCGAAGCTTAAATGCTAACCAGCTCAGTTC	*ppK*	HindIII
PPK-Rv1	ATCGGATCCTCTGATTCCGAACAGCGTG	*ppK*	BamHI
**For gene cloning**			
sprB-K6NcoI	AATCCATGGGAAATGTAATTATATACGGTATTAACTG	*sprB*	NcoI
sprB-K6PstI	TGCCTGCAGAAATATGCCTCAATGATTGAGCCAG	*sprB*	PstI
1770-SalIRv	GGAGTCGACCGTAAAAGTAGATAATATCTGTCG	*yobH*	SalI
SL1770-FW22	CCTGGATCCACAGCAACAGGCATACTAC	*yobH*	BamHI
**For gene deletions**			
SL1770H1P1	TCTAACTAAAAAAGAAAAGAGGTAGTAATGCGTTTGATCATT*TGTAGGCTGGAGCTGCTTCG*	*yobH*	
SL1770H2P2	ATTATTTATTGCCGCTTAGCCATTATCCACCACGGTGGCGAT*CATATGAATATCCTCCTTAG*	*yobH*	
sprB-H1P1	TCAAAAAAGGATAAATAAATGAGAAATGTAATTATATACGGT*TGTAGGCTGGAGCTGCTTCG*	*sprB*	

sprB-H2P2	ATGATAAAAAATATTTTAATTCATTCCTACCGCAATCGGTAA*CATATGAATATCCTCCTTAG*	*sprB*
SsinR-H1P1	TTTTATCAACGGAAGATAATGATGGAACTGATCAATAATCGT*TGTAGGCTGGAGCTGCTTCG*	*sinR*
SsinR-H2P2	AACCATTCCAAACGGGTTAATCATCATTCAGAAAATCAATTT*CATATGAATATCCTCCTTAG*	*sinR*
**For gene FLAG tagging**		
SL1770FLAG-F	TGTCCCATTTTCAGAAAAATCGCCACCGTGGTGGATAATGGC*GACTACAAAGACCATGACGG*	*yobH*
SL1770FLAG-R	AATTATTGGTGATAGTTTCACTGTGAAATTATTTATTGCCGC*CATATGAATATCCTCCTTAG*	*yobH*
**For chromosomal gene insertion**		
SL1770-FW22	CCTGGATCCACAGCAACAGGCATACTAC	*yobH*
1770FLAG-H2P2-C	TTATGAACAACAGCCAATATAGGGGAATTATTGGTGATAGTTTAATACGACTCACTATAGGGCG	*yobH*

Table S2. Primers used in this work. [1]Underlined letters indicate the respective restriction-enzyme site in the primer. [2]The sequences corresponding to the template plasmids pKD4 or pSUB11 (Table S1) are in italic letters. [3]RE, restriction enzyme for which a site was generated in the primer.

References

1. Hoiseth, S. K. & Stocker, B. A. D. Aromatic-dependent *Salmonella typhimurium* are non-virulent and effective as live vaccines. *Nature* **291,** 238–239 (1981).
2. Drecktrah, D., Knodler, L. A., Ireland, R. & Steele-Mortimer, O. The mechanism of *Salmonella* entry determines the vacuolar environment and intracellular gene expression. *Traffic* **7,** 39–51 (2006).
3. Bajaj, V., Lucas, R. L., Hwang, C. & Lee, C. A. Co-ordinate regulation of *Salmonella typhimurium* invasion genes by environmental and regulatory factors is mediated by control of *hilA* expression. *Mol. Microbiol.* **22,** 703–714 (1996).
4. Bustamante, V. H. *et al.* HilD-mediated transcriptional cross-talk between SPI-1 and SPI-2. *Proc. Natl. Acad. Sci. U. S. A.* **105,** 14591–6 (2008).
5. Martínez, L. C. *et al.* Integration of a complex regulatory cascade involving the SirA/BarA and Csr global regulatory systems that controls expression of the *Salmonella* SPI-1 and SPI-2 virulence regulons through HilD. *Mol. Microbiol.* **80,** 1637–1656 (2011).
6. Pérez-Morales, D. *et al.* The transcriptional regulator SsrB is involved in a molecular switch controlling virulence lifestyles of *Salmonella*. *PLOS Pathog.* **13,** e1006497 (2017).
7. Martínez-Flores, I. *et al. In silico* clustering of *Salmonella* global gene expression data reveals novel genes co-regulated with the SPI-1 virulence genes through HilD. *Sci. Rep.* **6,** 37858 (2016).
8. Ferenci, T. *et al.* Genomic sequencing reveals regulatory mutations and recombinational events in the widely used MC4100 lineage of *Escherichia coli* K-12. *J. Bacteriol.* **191,** 4025–9 (2009).
9. Bustamante, V. H. *et al.* PerC and GrlA independently regulate Ler expression in enteropathogenic *Escherichia coli*. *Mol. Microbiol.* **82,** 398–415 (2011).
10. Brosius, J. Plasmid vectors for the selection of promoters. *Gene* **27,** 151–160 (1984).
11. Mayer, M. P. A new set of useful cloning and expression vectors derived from pBlueScript. *Gene* **163,** 41–46 (1995).
12. Banda, M. M. *et al.* HilD and PhoP independently regulate the expression of *grhD1*, a novel gene required for *Salmonella* Typhimurium invasion of host cells. *Sci. Rep.* **8,** 4841 (2018).
13. Datsenko, K. A. & Wanner, B. L. One-step inactivation of chromosomal genes in *Escherichia coli* K-12 using PCR products. *Proc. Natl. Acad. Sci. U. S. A.* **97,** 6640–5 (2000).
14. Uzzau, S., Figueroa-Bossi, N., Rubino, S. & Bossi, L. Epitope tagging of chromosomal genes in *Salmonella*. *Proc. Natl. Acad. Sci. U. S. A.* **98,** 15264–9 (2001).
15. Husseiny, M. I. & Hensel, M. Rapid method for the construction of *Salmonella enterica* serovar Typhimurium vaccine carrier strains. *Infect. Immun.* **73,** 1598–605 (2005).
